# Supplementary material for: Blockage of Store-Operated Ca2+ Influx by Synta66 is Mediated by Direct Inhibition of the Ca2+ Selective Orai1 Pore
Source: Cancers (Basel). 2020 Oct 6;12(10):2876. doi: 10.3390/cancers12102876 (PMC7600887; doi:10.3390/cancers12102876)
Supplement: Supplementary file 1 [file cancers-12-02876-s001.pdf]

cancers

MDPI

Supplementary Materials

A Ca^{2+} selective Orai1 pore is required for Synta66 mediated store-operated channel inhibition

Linda Waldherr, Adela Tiffner, Deepti Mishra, Matthias Sallinger, Romana Schober, Irene Frischauf, Tony Schmidt, Verena Handl, Peter Sagmeister, Manuel Köckinger, Isabella Derler, Muammer Üçal, Daniel Bonhenry*, Silke Patz* and Rainer Schindl*

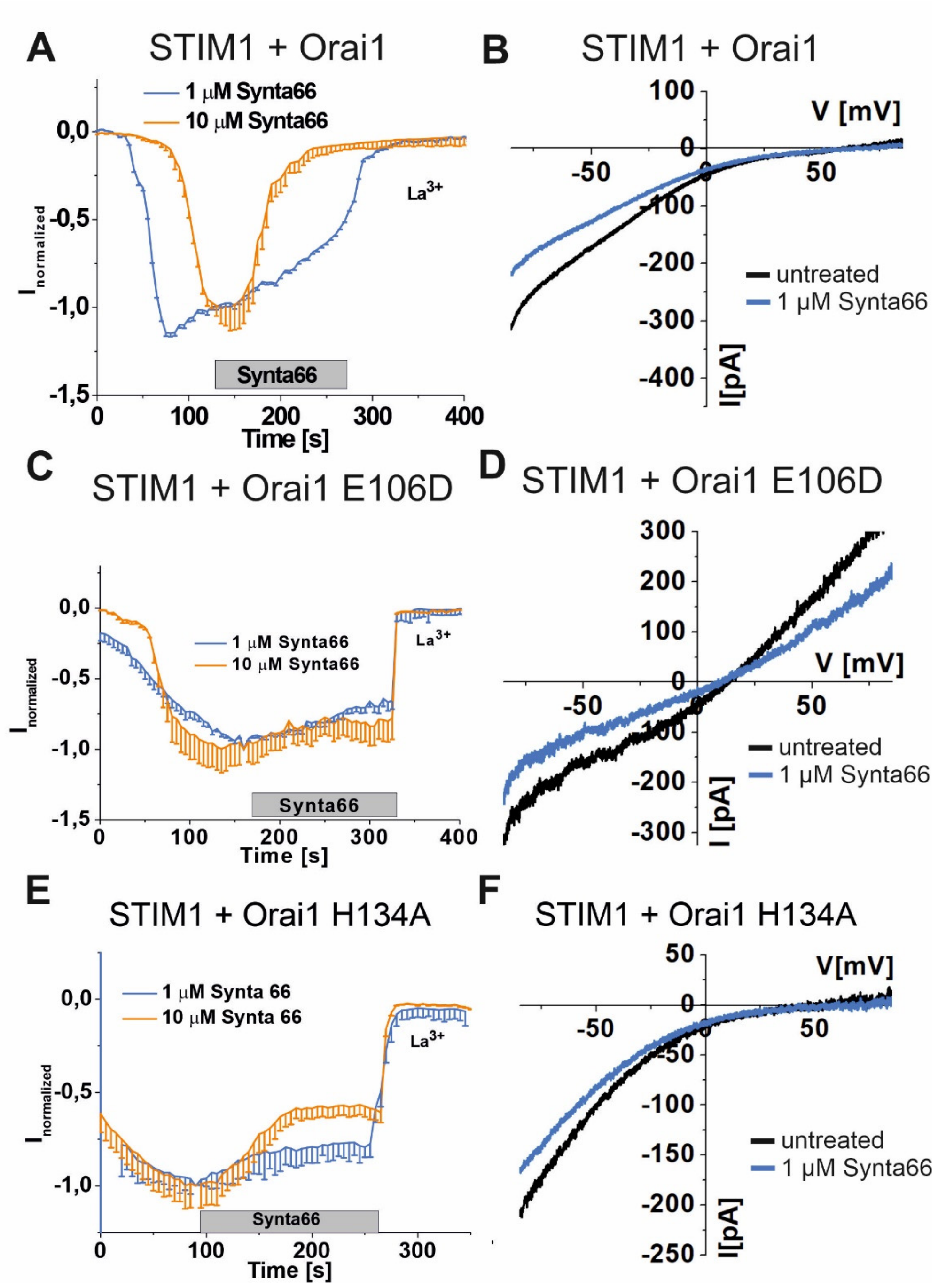

Figure S1. The inhibitory action of 1 and 10 μM Synta66 in Orai1 wild-type and mutations (**a, c, e**) Time course of normalized whole-cell inward rectifying currents at −86 mV, maximally activated upon passive store depletion of HEK293 cells were recorded with co-expressing STIM1 and Orai1 (a), Orai1 E106D (c) or Orai1 H134A (e), upon perfusion of 1 μM Synta66 and subsequent block by 10 μM La^{3+} (n = 6-10 cells, from at least two individual transfections). (**b, d, f**) Corresponding I/V relationships of STIM1 and Orai1 (**b**), Orai1 E106D (**d**) or Orai1 H134A (f) after maximal store-operated activation and upon addition of 1 μM Synta66 (blue).

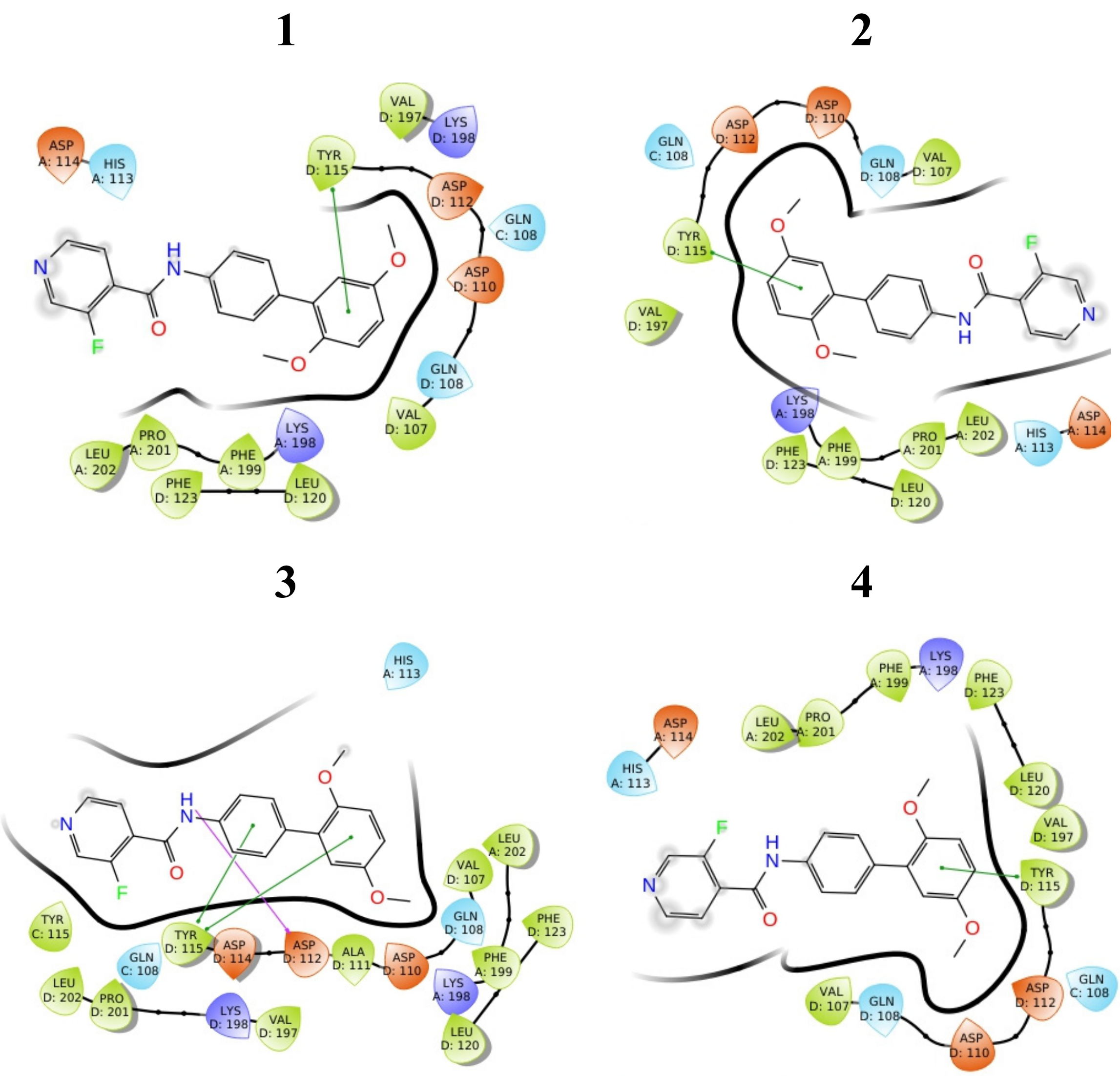

Number	Docking score
#1	-3.622
#2	-3.603
#3	-3.581
#4	-3.557

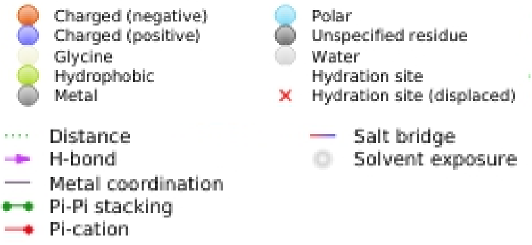

Figure S2. Docking positions of Synta66 with Orai: Different docking positions for Synta66 in the Orai1 pore and their corresponding glide scores. The Interaction Diagram (LID) shows the four best scoring poses of Synta66 with Orai1.

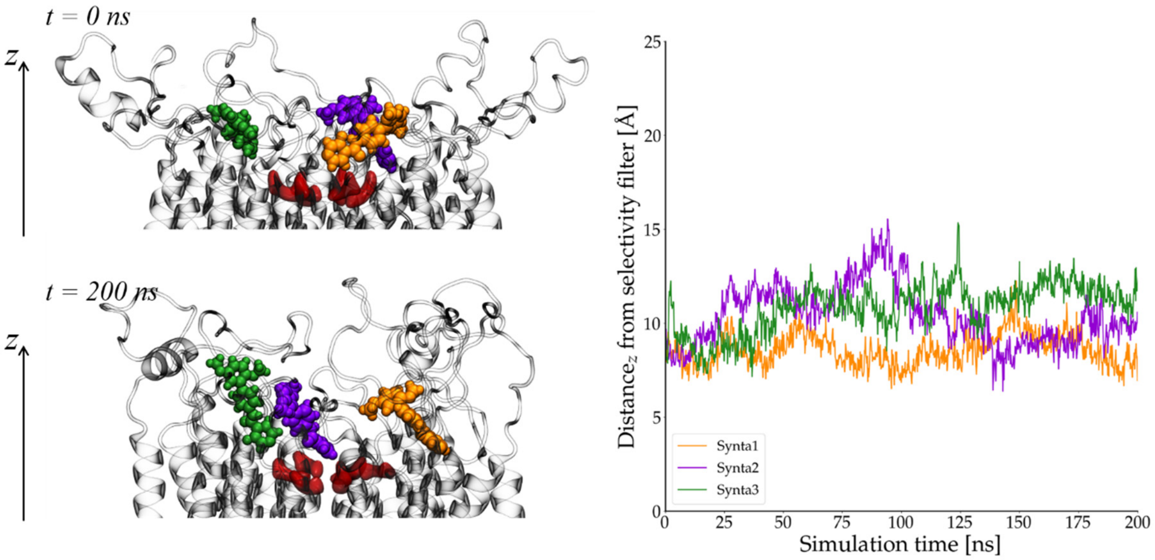

Figure S3. Synta66 remains its interaction to Orai1 at the docked loop1/loop3 pose 1:

Upper Left: Starting configuration with three molecules of Synta66 present at the entry of the pore. The protein is represented as a gray glassy ribbon with the selectivity filter highlighted in red. Individual molecules of Synta66 are represented in orange, violet and green.

Lower left: Configuration of the system after 200 ns.

Right: Projection of the distance along the z-axis of the distances between the center-of-mass of each molecule of Synta66 to the selectivity filter as a function of the simulation time. Evolution for individual molecules of Synta66 are shown in orange, violet and green concomitantly with the representative snapshots presented on the left.

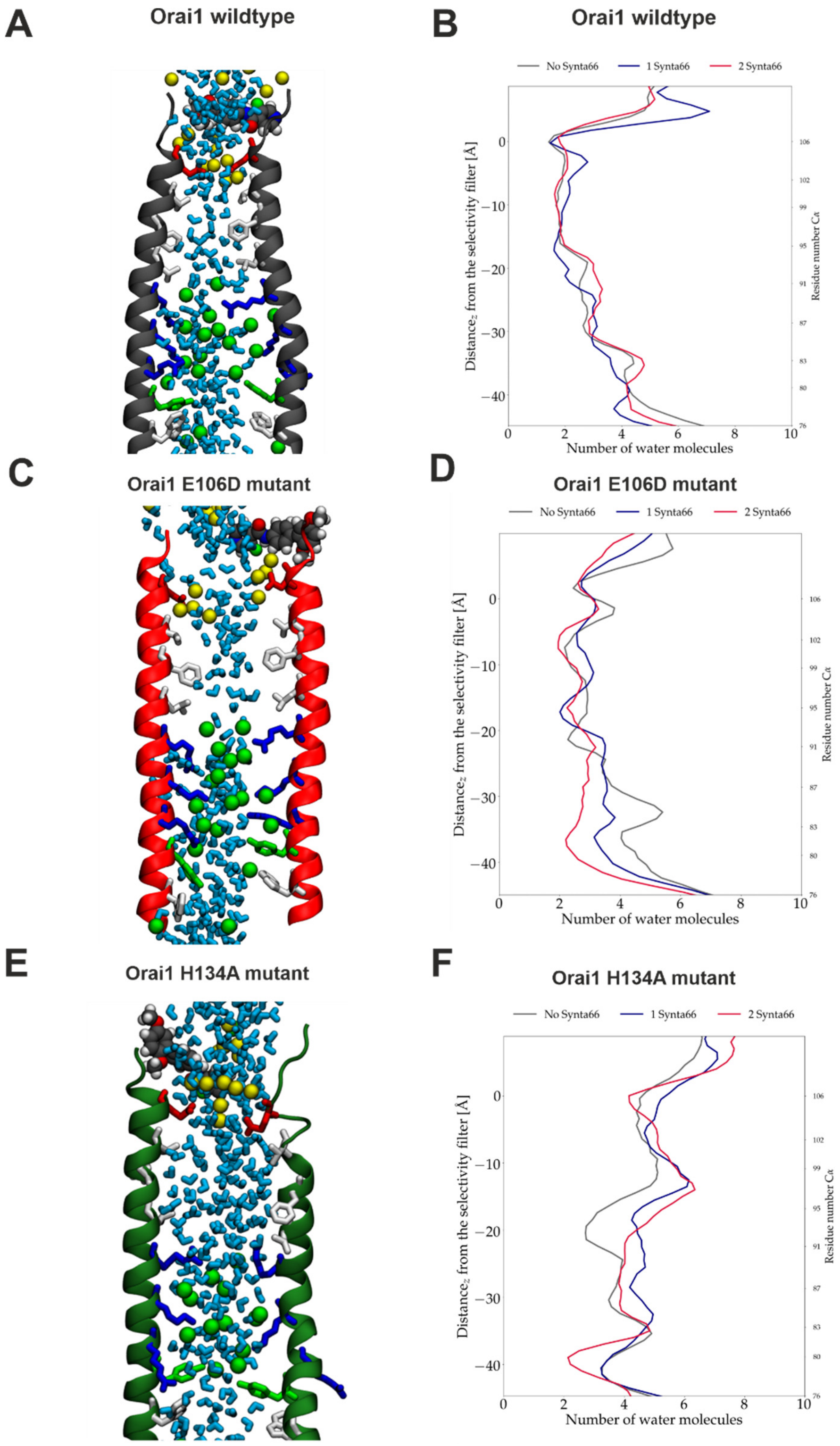

Figure S4. Hydration of the Orai1 wild-type and mutant pore: (**a, c, e**) Representative snap shots of the equilibrated part of 250-ns-long molecular dynamics simulations for (A) wild-type Orai1, (C) Orai1-E106D and (E) Orai1-H134A illustrates the pore-forming TM1 helices (2 out of 6 TM1 helices) and pore-lining residues from Glu106 to Phe76. Water molecules, and cations (yellow ball), and Cl- (green ball) and a single Synta66 compound are shown in the respective pores; (**b, d, f**) Average number of water molecules (over the last 50 ns of respective simulations) lining the pores of (B) wild-type Orai1, (D) Orai1-E106D and (F) Orai1-H134A with no Synta66 (black), 1 Synta66 (blue) and two Synta66 molecules (red).

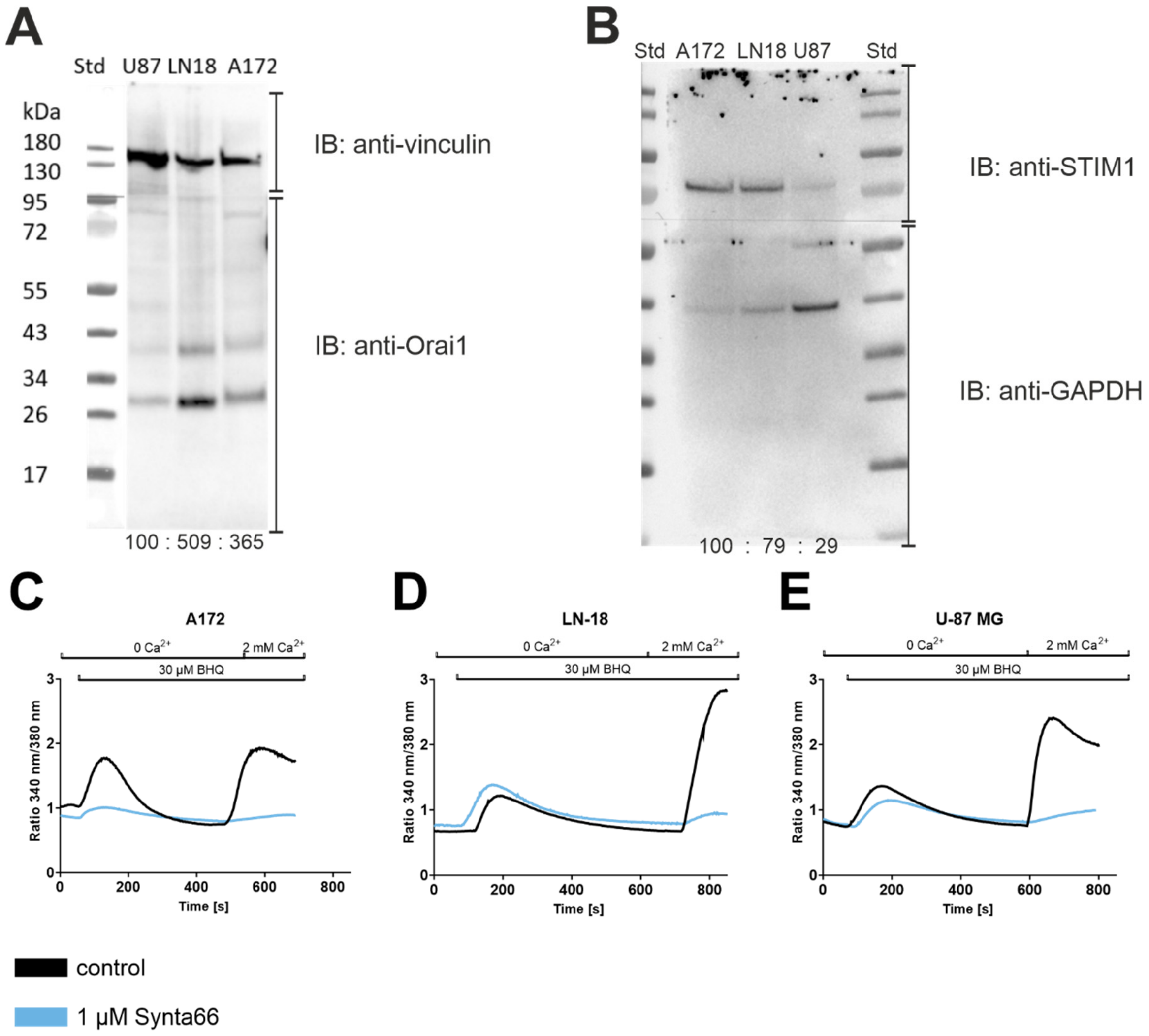

Figure S5. Expression of STIM and Orai in GBM cell lines and Inhibition of SOCE in GBM cell lines with 1 µM Synta66: (a) Western Blot for detection of endogenously expressed Orai1 in lysates of A172, LN-18 and U-87 MG cells. Lysates were deglycosylated in order to achieve a single Orai1 band as in (50). (**b**) Western Blot for detection of endogenously expressed STIM1 in lysates of A172 (1), LN-18 (2) and U-87 MG cells (3). **(c-e)**: Representative time course experiments of cytosolic Ca2+ measurements in Fura-2 AM loaded GBM cell lines. Cells are monitored initially in a Ca^{2+} free extracellular solution followed by application of 30 µM BHQ and addition of 2mM Ca2+ to monitor SOCE. Analogous experiments with pre-treatment of 1 µM Synta66 immediately before the start of the experiment was used. Remaining SOCE in A172 at 2.8 ± 0.3 %, in LN-18 cells 6.2 ± 0.4 % and in U-87 MG cells 14.8 ± 0.6 %.

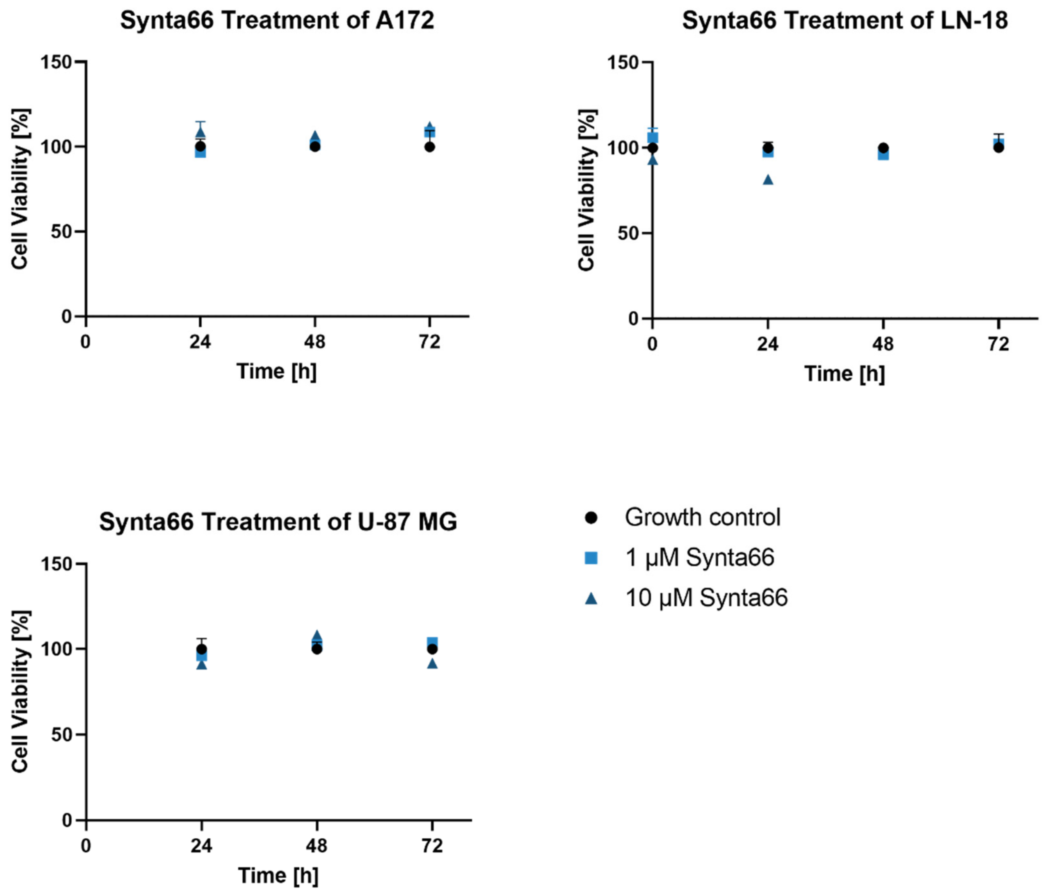

Figure S6. Cell Viability with Synta66. Effect on cell viability of Synta66 treatment (1 and 10 μM) was observed in GBM cell lines over 72 h by readout with MTS assay. Results shown as mean ± SEM, n=6 from two independent experiments.

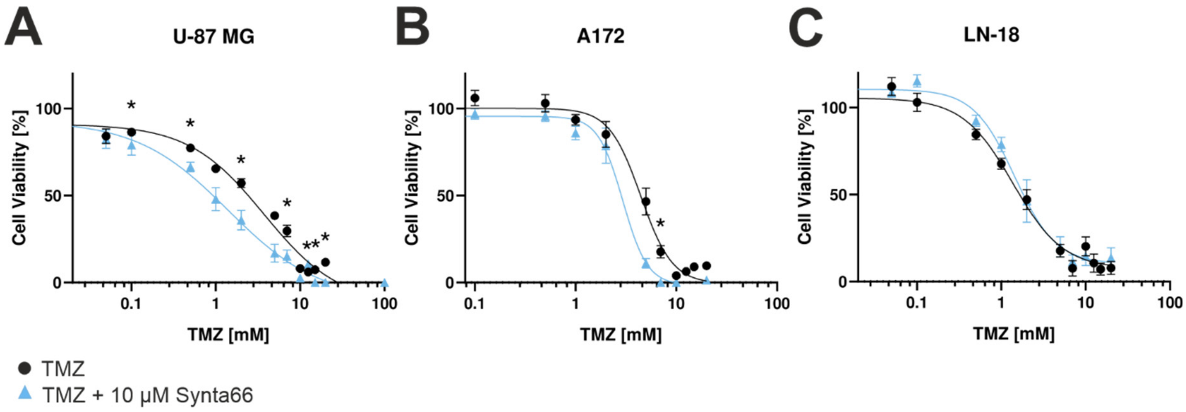

Figure S7. Synergistic effects of Synta66 on TMZ treatment: (**a-c**) Cell viability after 72 h treatment with different TMZ concentrations ± 10 μM Synta66 was observed in GBM cell lines via MTS assay. Cells were treated with fresh solutions of TMZ, in order to keep the DMSO concentration <0.1 %. Results shown as mean ± SEM (n = 6 from two independent experiments, *: p-value < 0.05). In (**a**): IC_{50}: TMZ alone = 3.7 mM, TMZ+10 μM Synta66 = 1.4 mM, $p < 0.05$). In (**b**) IC_{50} TMZ alone = 4.4 mM and TMZ+10 μM Synta66 = 2.9 mM.

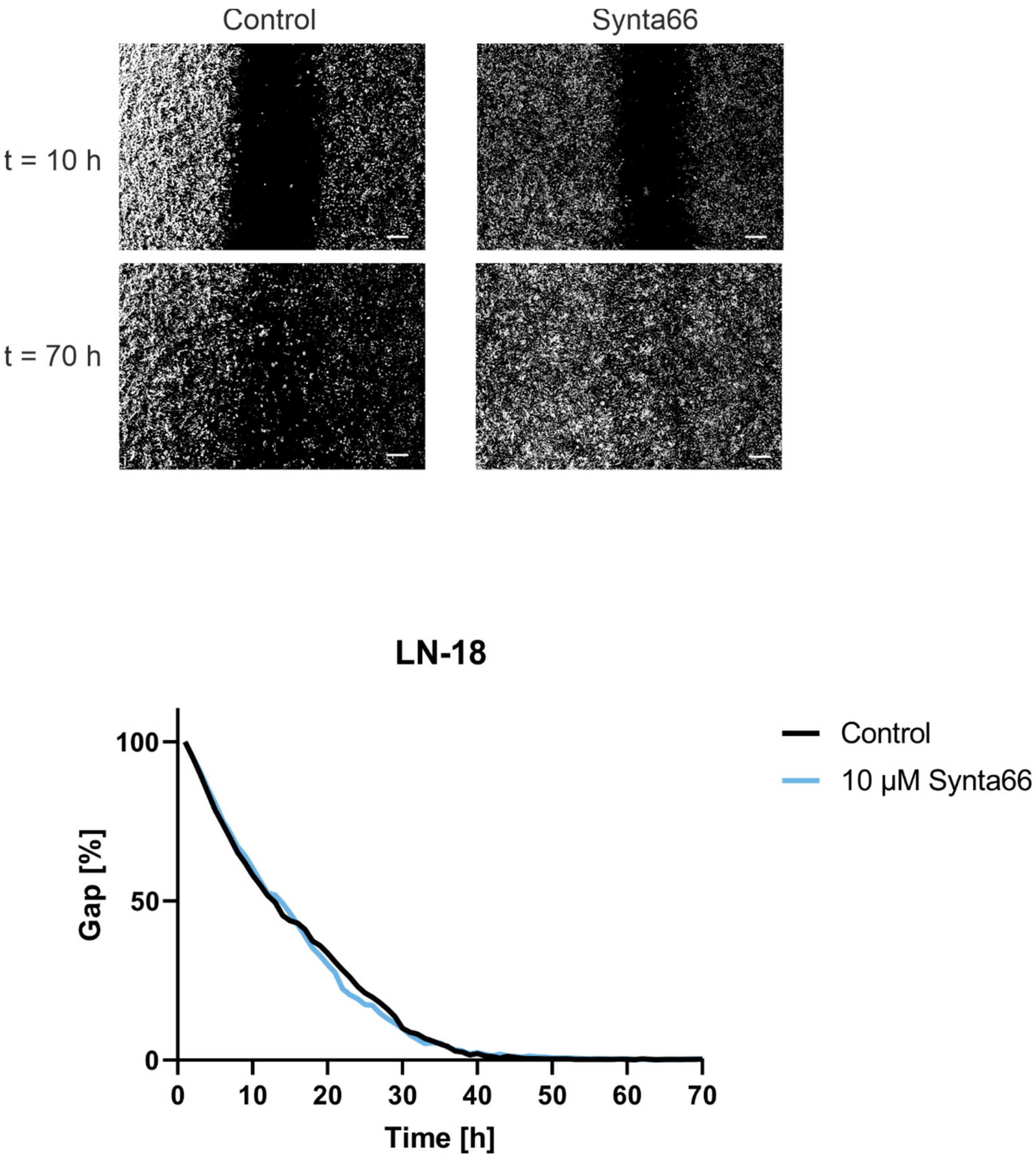

Figure S8. Migration assay with Synta66 treatment in LN-18 cells: Time course experiments show mean values of normalized gap distances for control and Synta66 treated LN-18 cells. Representative images of scratch in cell layer at 10 and 70 h. (n=40-43).
